# Updated overall survival from the MONALEESA-3 trial in postmenopausal women with HR+/HER2− advanced breast cancer receiving first-line ribociclib plus fulvestrant

**DOI:** 10.1186/s13058-023-01701-9

**Published:** 2023-08-31

**Authors:** P. Neven, P. A. Fasching, S. Chia, G. Jerusalem, M. De Laurentiis, S.-A. Im, K. Petrakova, G. V. Bianchi, M. Martín, A. Nusch, G. S. Sonke, L. De la Cruz-Merino, J. T. Beck, J. P. Zarate, Y. Wang, A. Chakravartty, C. Wang, D. J. Slamon

**Affiliations:** 1 Multidisciplinary Breast Centre, Universitair Ziekenhuis Leuven, Herestraat 49, 3000 Leuven, Belgium; 2 University Hospital Erlangen, Friedrich-Alexander University Erlangen-Nuremberg, Erlangen, Germany; 3 British Columbia Cancer Agency, Vancouver, BC Canada; 4 CHU Liege and Liège University, Liège, Belgium; 5 Istituto Nazionale Tumori IRCCS “Fondazione G. Pascale”, Naples, Italy; 6 Cancer Research Institute, Seoul National University Hospital, Seoul National University College of Medicine, Seoul, Republic of Korea; 7 Masaryk Memorial Cancer Institute, Brno, Czech Republic; 8 Fondazione Istituto di Ricovero e Cura a Carattere Scientifico, Istituto Nazionale dei Tumori, Milan, Italy; 9 Instituto de Investigación Sanitaria Gregorio Marañon, Centro de Investigación Biomédica en Red de Cáncer, Grupo Español de Investigación en Cáncer de Mama, Universidad Complutense, Madrid, Spain; 10 Practice for Hematology and Internal Oncology, Velbert, Germany; 11 Netherlands Cancer Institute/Borstkanker Onderzoek Groep Study Center, Amsterdam, The Netherlands; 12 Hospital Universitario Virgen Macarena, Seville, Spain; 13 Highlands Oncology, Springdale, AR USA; 14 Novartis Pharmaceuticals Corporation, East Hanover, NJ USA; 15 Novartis Pharma AG, Basel, Switzerland; 16 David Geffen School of Medicine at UCLA, Los Angeles, CA USA

**Keywords:** Ribociclib, CDK4/6 inhibitor, Advanced breast cancer, Overall survival, First line

## Abstract

**Background:**

The phase III MONALEESA-3 trial included first- (1L) and second-line (2L) patients and demonstrated a significant overall survival (OS) benefit for ribociclib + fulvestrant in patients with hormone receptor–positive, human epidermal growth factor receptor 2–negative (HR+/HER2−) advanced breast cancer (ABC) in the final protocol-specified and exploratory (longer follow-up) OS analyses. At the time of these analyses, the full OS benefit of 1L ribociclib was not completely characterized because the median OS (mOS) was not reached. As CDK4/6 inhibitor (CDK4/6i) + endocrine therapy (ET) is now a preferred option for 1L HR+/HER2− ABC, we report an exploratory analysis (median follow-up, 70.8 months; 14.5 months longer than the prior analysis) to fully elucidate the OS benefit in the MONALEESA-3 1L population.

**Methods:**

Postmenopausal patients with HR+/HER2− ABC were randomized 2:1 to 1L/2L fulvestrant + ribociclib or placebo. OS in 1L patients (de novo disease or relapse > 12 months from completion of [neo]adjuvant ET) was assessed by Cox proportional hazards model and Kaplan–Meier methods. Progression-free survival 2 (PFS2) and chemotherapy-free survival (CFS) were analyzed. MONALEESA-3 is registered with ClinicalTrials.gov (NCT02422615).

**Results:**

At data cutoff (January 12, 2022; median follow-up time, 70.8 months), mOS was 67.6 versus 51.8 months with 1L ribociclib versus placebo (hazard ratio (HR) 0.67; 95% CI 0.50–0.90); 16.5% and 8.6% of ribociclib and placebo patients, respectively, were still receiving treatment. PFS2 (HR 0.64) and CFS (HR 0.62) favored ribociclib versus placebo. Among those who discontinued treatment, 16.7% and 35.0% on ribociclib or placebo, respectively, received a subsequent CDK4/6i. No new safety signals were observed.

**Conclusions:**

This analysis of MONALEESA-3 reports the longest mOS thus far (67.6 months) for 1L patients in a phase III ABC trial. These results in a 1L population show that the OS benefit of ribociclib was maintained through extended follow-up, further supporting its use in HR+/HER2− ABC.

**Supplementary Information:**

The online version contains supplementary material available at 10.1186/s13058-023-01701-9.

## Introduction

The addition of CDK4/6 inhibitors (CDK4/6is) to endocrine therapy (ET) has greatly improved outcomes for patients with hormone receptor–positive (HR+)/human epidermal growth factor receptor 2–negative (HER2−) advanced breast cancer (ABC) [1]. Accordingly, CDK4/6i + ET is now considered a preferred first-line treatment option [2, 3]. All three approved CDK4/6is (ribociclib, palbociclib, and abemaciclib) have demonstrated a significant progression-free survival (PFS) benefit compared with ET alone when used in the first-line setting [4–8]. Recently, data from final prespecified overall survival (OS) analyses of CDK4/6is + ET in the first-line setting have become available.

First-line palbociclib in combination with the nonsteroidal aromatase inhibitor (NSAI) letrozole failed to demonstrate a statistically significant improvement in OS over letrozole alone in postmenopausal patients in the PALOMA-2 trial (median OS [mOS], 53.9 vs. 51.2 months [hazard ratio (HR) 0.96; 95% CI 0.78–1.18; *P* = 0.34]) [9]. The second interim OS analysis of MONARCH-3 demonstrated a median OS of 67.1 versus 54.5 months (HR 0.754; 95% CI 0.584–0.974; *P* = 0.0301) for first-line abemaciclib + NSAI versus NSAI; however, prespecified criteria for statistical significance were not met, and final OS results have not yet been reported [10]. These agents have also been studied in combination with fulvestrant in patients (any menopausal status) previously treated with ET, including subsets of patients with early relapse being treated for the first time for ABC, and OS results are available. In PALOMA-3, palbociclib + fulvestrant failed to demonstrate a statistically significant OS benefit in the final prespecified (HR 0.81; 95% CI 0.64–1.03; *P* = 0.09) or extended follow-up analysis (HR 0.81; 95% CI 0.65–0.99) [11, 12]. The MONARCH-2 trial demonstrated a significant OS benefit for abemaciclib + fulvestrant versus fulvestrant alone (mOS, 46.7 vs. 37.3 months [HR 0.757; 95% CI 0.61–0.95; *P* = 0.01]) [13].

Ribociclib has demonstrated a significant PFS and OS benefit in all three of its pivotal phase III clinical trials in patients with HR+/HER2− ABC. In MONALEESA-2, first-line ribociclib + letrozole in postmenopausal women demonstrated a significant 12.5-month improvement over letrozole alone, with an mOS of 63.9 versus 51.4 months (HR 0.76; 95% CI 0.63–0.93; *P* = 0.008) [14]. In MONALEESA-7, a significant OS benefit was observed with first-line ribociclib + NSAI versus NSAI alone (mOS, not reached [NR] vs. 40.7 months [HR 0.70; 95% CI 0.50–0.98]) in pre/perimenopausal women; in addition, an exploratory analysis with extended follow-up reported an mOS of 58.7 versus 47.7 for ribociclib + NSAI versus NSAI alone (HR 0.798; 95% CI 0.62–1.04) [15, 16]. MONALEESA-3 studied first-line (no prior treatment for ABC, including those who relapsed > 12 months after the end of [neo]adjuvant ET [late relapse] or patients with de novo advanced/metastatic disease [no prior exposure to ET]) or second-line (relapse ≤ 12 months from completion of [neo]adjuvant ET [early relapse] or progression on first-line ET for ABC) ribociclib + fulvestrant versus fulvestrant alone in postmenopausal patients. To date, MONALEESA-3 is the only trial of a CDK4/6i with fulvestrant as an ET partner to report OS results for a first-line population that included patients with de novo and late relapse disease. In the final prespecified analysis, a significant OS benefit was observed with ribociclib + fulvestrant over fulvestrant alone; however, at the time of this analysis, the mOS was not reached in the ribociclib arm in the intent-to-treat (ITT) population (mOS, NR vs. 40.0 months [HR 0.72; 95% CI 0.57–0.92; *P* = 0.00455]) nor was it reached in the first-line (de novo or late relapse) subgroup (mOS, NR vs. 45.1 months [HR 0.70; 95% CI 0.48–1.02]). In the second line (early relapse or one prior ET for ABC), the mOS was 40.2 versus 32.5 months (HR 0.73; 95% CI 0.53–1.00) for ribociclib + fulvestrant versus fulvestrant alone [17]. An exploratory analysis with an additional 16.9 months of follow-up resulted in a more than 1-year improvement in mOS with ribociclib + fulvestrant over fulvestrant alone for the ITT population (mOS, 53.7 vs. 41.5 months; HR 0.73; 95% CI 0.59–0.90); the mOS was still not reached for ribociclib + fulvestrant in the first-line (de novo or late relapse) population (mOS, NR vs. 51.8 months for fulvestrant alone [HR 0.64; 95% CI 0.46–0.88]). With this longer follow-up, the mOS for ribociclib + fulvestrant in the second-line (early relapse or one prior ET for ABC) population remained consistent with prior results (mOS, 39.7 vs. 33.7 months for fulvestrant alone [HR 0.78; 95% CI 0.59–1.04]) [18].

As the combination of a CDK4/6i + ET is the recommended first-line option for patients with HR+/HER2− ABC, it is highly clinically relevant to understand the OS benefits of ribociclib + fulvestrant in this population [2, 3]. Therefore, this exploratory OS analysis with an extended follow-up time (median, 70.8 months) was undertaken to elucidate the full impact of first-line use of ribociclib + fulvestrant and to describe the updated results of this combination when used in the second-line setting.

## Methods

### Study design

Details of the MONALEESA-3 trial have been described previously [8, 17]. Briefly, patients were randomly assigned (2:1) to receive either oral ribociclib (600 mg/day on a 3-weeks-on, 1-week-off schedule) or matching placebo. Both groups received intramuscular fulvestrant (500 mg, day 1 of every 28-day cycle, with an additional dose on day 15 of cycle 1). Randomization was stratified by the presence or absence of liver or lung metastases and prior ET (no prior ET for ABC vs. up to one line of ET for ABC). All patients and investigators, including those who administered treatment, assessed outcomes, and analyzed data, were blinded to the trial group assignments. Crossover was not allowed until the protocol-prespecified final OS analysis was completed. After the final OS analysis, patients and investigators were unblinded and patients in the placebo arm who were still receiving study treatment were given the option to switch to ribociclib.

### Participants

Men and postmenopausal women aged ≥ 18 years, with histologically or cytologically confirmed HR+/HER2− ABC (locoregionally recurrent or metastatic and not amenable to curative therapy) were eligible for the study. An Eastern Cooperative Oncology Group performance status of 0 or 1 and measurable disease according to Response Evaluation Criteria in Solid Tumors version 1.1 or at least one predominantly lytic bone lesion was required.

Patients receiving treatment in the first-line setting were those with no prior treatment for ABC, including those who relapsed > 12 months after the end of (neo)adjuvant ET (late relapse) or patients with de novo advanced/metastatic disease (no prior exposure to ET). Patients characterized as receiving treatment in the second-line setting included those who relapsed ≤ 12 months from completion of (neo)adjuvant ET (early relapse) or progressed on first-line ET for ABC. Despite receiving treatment in the advanced setting for the first time, patients with early relapse were analyzed with the second-line population of patients due to having a more similar prognosis to this population compared with the otherwise defined first-line population in the study. Patients who had received previous chemotherapy for advanced disease or any previous treatment with fulvestrant or a CDK4/6i were not included.

### Endpoints

The final prespecified analyses of the primary endpoint of investigator-assessed PFS and the secondary endpoint of OS, as well as the extended follow-up (median, 56.3 months) analysis of MONALEESA-3, have been reported previously [8, 17]. OS, a protocol-specified secondary endpoint, was defined as the time from randomization to death from any cause. Chemotherapy-free survival (CFS), time to chemotherapy (TTC), and PFS2 were additional exploratory endpoints. CFS was defined as the time from randomization to the beginning of first subsequent chemotherapy or death. TTC was defined as the time from randomization to the beginning of the first subsequent chemotherapy following discontinuation of study treatment. While CFS factors in deaths as events, TTC censors deaths. PFS2 was defined as the time from randomization to the first documented disease progression (as reported by the investigator) while the patient was receiving next-line therapy or death from any cause, whichever occurred first.

Survival follow-up continued for patients who discontinued study treatment. Adverse events (AEs) were monitored and graded according to the Common Terminology Criteria for Adverse Events (version 4.03) [8]. Safety follow-up was conducted for ≥ 30 days after the patients’ last study treatment dose.

### Statistical analysis

In this exploratory analysis of OS, mOS and OS rates were estimated using the Kaplan–Meier method. The HR for OS was estimated using a Cox proportional hazards model. Patients without events were censored at the date they were last known to be alive. Analyses were performed on the data in the overall trial population, on patients receiving first-line therapy (de novo or late relapse), and on patients receiving second-line therapy (early relapse or one prior ET for ABC). In addition to the OS analyses, in patients receiving first-line therapy, CFS, TTC, and PFS2 were analyzed using the Cox proportional hazards model and Kaplan–Meier method. In the current extended follow-up (data cutoff, January 12, 2022), a sufficient number of events was reported in the first-line ribociclib arm to provide an estimate of mOS. For patients receiving first-line therapy, the rank-preserving structural-failure time model was used as a sensitivity analysis on OS to determine the effects of crossover and administration of subsequent CDK4/6is in the placebo group.

## Results

### Patient disposition

Overall, 726 postmenopausal women were randomly assigned between June 18, 2015, and June 10, 2016: 484 to the ribociclib arm and 242 to the placebo arm. The baseline characteristics of the patients were included in the previously published analyses [8]. A total of 53 of 484 patients (11.0%) in the ribociclib arm and 15 of 242 patients (6.2%) in the placebo arm were still receiving study treatment at the cutoff date (January 12, 2022) for this analysis. The median duration of follow-up for the trial (from randomization to data cutoff) was 70.8 months (minimum, 67.3 months).

In the first-line (de novo or late relapse) subgroup, 237 patients were randomized to the ribociclib arm and 128 to the placebo arm. A total of 39 of 237 patients (16.5%) in the ribociclib arm and 11 of 128 patients (8.6%) in the placebo arm were still receiving study treatment at the cutoff date. Following the final OS analysis, 2 patients (1.6%) in the placebo arm elected to cross over and received at least one dose of ribociclib. A total of 133 patients (56.1%) in the ribociclib arm and 101 patients (78.9%) in the placebo arm discontinued first-line treatment due to disease progression. AEs (7.7%), patient/guardian decisions (7.1%), physician decision (6.8%), death (0.3%), and protocol deviation (0.3%) were other reasons for discontinuing first-line treatment (Additional file 1: Table S1).

### Overall survival

**Fig. 1:**
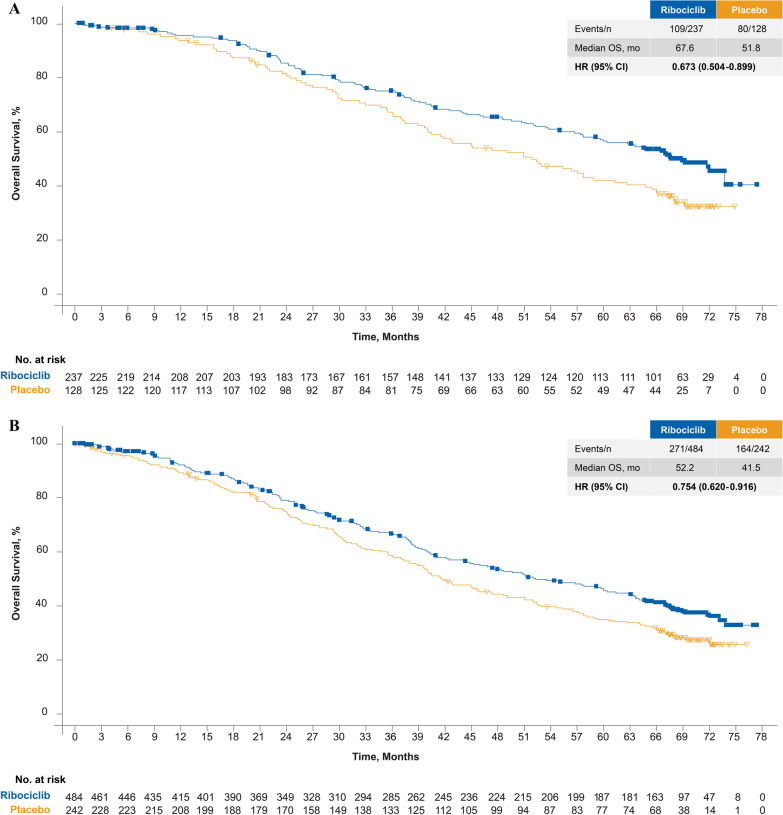

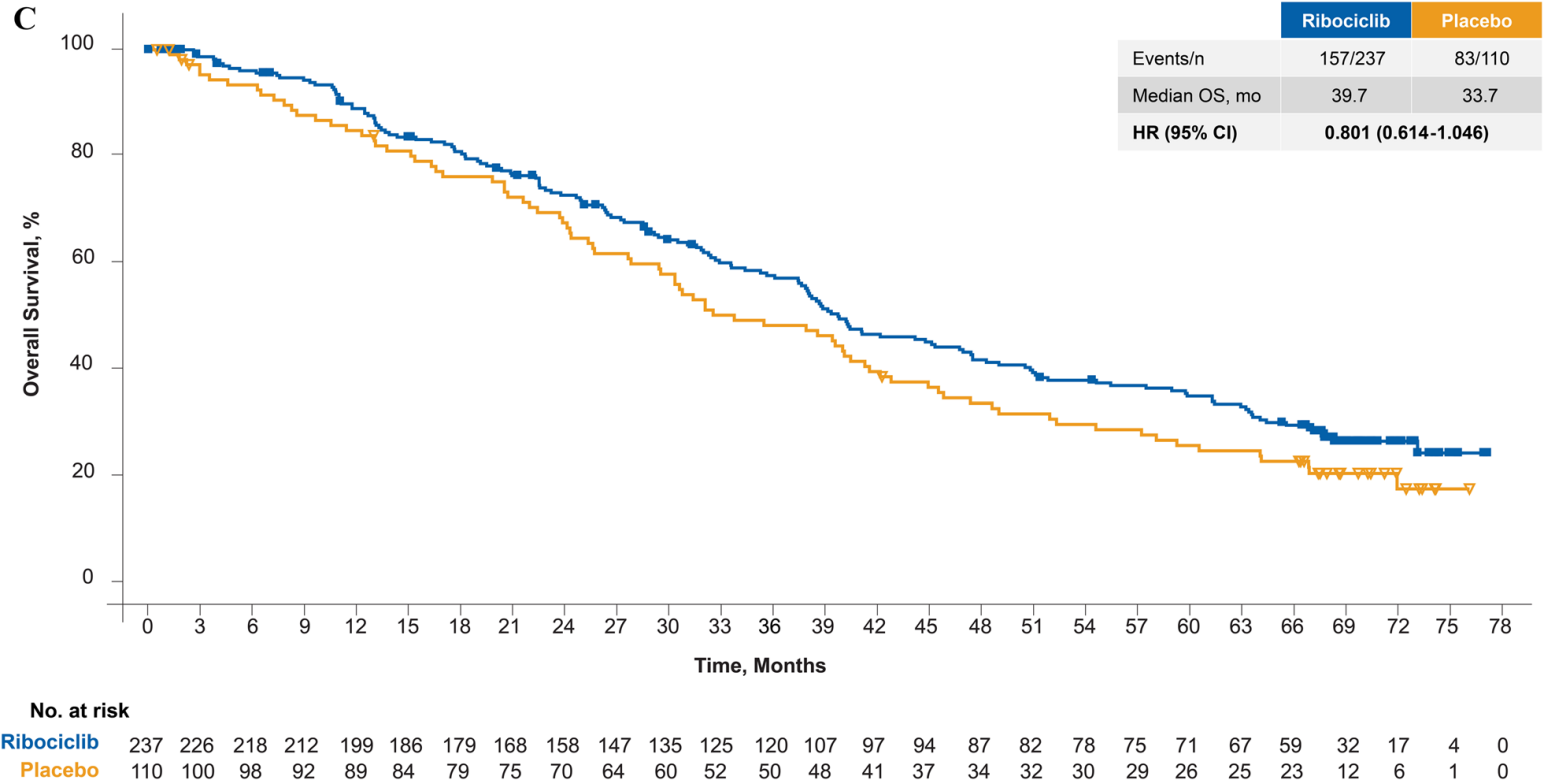
**A** Overall survival in patients who received ribociclib plus fulvestrant as first-line therapy. **B** Overall survival in ITT population. **C** Overall survival in patients who received ribociclib plus fulvestrant as second-line therapy

In patients receiving first-line therapy, ribociclib + fulvestrant resulted in a significant OS benefit (mOS, 67.6 months; 95% CI 59.6–NE) versus fulvestrant alone (51.8 months; 95% CI 40.4–61.2 months), with a 33% relative reduction in risk of death with ribociclib treatment (HR 0.67; 95% CI 0.50–0.90) (Fig. 1A). Kaplan–Meier–estimated 5-year survival rates were 56.5% (95% CI 49.5–62.9%) and 42.1% (95% CI 33.2–50.7%) for ribociclib and placebo, respectively, in this population.

When OS was assessed by metastatic presentation (ET naive or late relapse) in patients receiving first-line therapy, ribociclib + fulvestrant (n = 138; mOS, 67.4 months) demonstrated an OS benefit versus fulvestrant alone (n = 73; mOS, 50.9 months) in patients with ET-naive disease (HR 0.67; 95% CI 0.46–0.98). Likewise, ribociclib + fulvestrant (n = 99; mOS, 71.6 months) demonstrated a trend toward clinically meaningful OS benefit versus fulvestrant alone (n = 55; mOS, 52.3 months) in those with late relapse (HR 0.68; 95% CI 0.43–1.05). These results were consistent with a prior analysis on de novo and late-relapse patients using an earlier data cutoff date (October 30, 2020) [19].

Furthermore, similar to the previous OS analysis [18], ribociclib was associated with an OS benefit in the overall trial population and second-line populations (early relapse or one prior ET for ABC). In the overall trial population, ribociclib + fulvestrant demonstrated a significant OS benefit with an mOS of 52.2 months in the ribociclib arm (n = 484) versus 41.5 months in the placebo arm (n = 242; HR 0.75; 95% CI 0.62–0.92; Fig. 1B), with Kaplan–Meier–estimated 5-year survival rates of 45.6% (95% CI 40.8–50.2%) and 35.0% (95% CI 28.8–41.2%) for ribociclib and placebo, respectively. For the second-line population (early relapse or one prior ET for ABC), the mOS was 39.7 months in the ribociclib arm (n = 237) versus 33.7 months in the placebo arm (n = 110; HR 0.80; 95% CI 0.61–1.05; Fig. 1C); the Kaplan–Meier–estimated 5-year survival rates were 34.9% (95% CI 28.6–41.4%) for ribociclib and 25.7% (95% CI 17.7–34.4%) for the placebo arm.

### Subsequent therapy in patients in the first-line subgroup

Of the patients treated in the first-line setting (de novo or late relapse) who discontinued study treatment, 162 of 198 patients (81.8%) in the ribociclib arm and 105 of 117 patients (89.7%) in the placebo arm received subsequent antineoplastic therapy (Table 1). The most common subsequent therapies were hormonal therapy alone (35.9% and 25.6%), hormonal therapy + targeted/other therapy (22.7% and 34.2%), and chemotherapy alone or in combination with other/hormonal therapy (20.7% and 29.1%) for the ribociclib and placebo arms, respectively (Table 1). Following discontinuation from the study, 33 of 198 patients (16.7%) in the ribociclib arm versus 41 of 117 patients (35.0%) in the placebo arm received a CDK4/6i at any time (Table 1). After adjustment for the subsequent CDK4/6i treatment using rank-preserving structural-failure time analysis, the mOS in the placebo arm was estimated to be 50.4 months (HR 0.62; 95% CI 0.43–0.88), which corresponded to what was observed in the main analysis (51.8 months [HR 0.67; 95% CI 0.50–0.89]).

**Table 1:** Subsequent therapy in patients who received ribociclib plus fulvestrant as first-line therapy

Parameter, n (%)	Ribociclib + Fulvestrant n = 237	Placebo + Fulvestrant n = 128
Patients who discontinued study treatment	198 (83.5)	117 (91.4)
Patients who received first subsequent antineoplastic therapy	162 (81.8)	105 (89.7)
Chemotherapy alone	25 (12.6)	19 (16.2)
Chemotherapy + hormonal or other therapy^a^	16 (8.1)	15 (12.8)
Hormonal therapy alone	71 (35.9)	30 (25.6)
Hormonal therapy + targeted or other therapy^b^	45 (22.7)	40 (34.2)
Targeted therapy alone or other therapy	5 (2.5)	1 (0.9)
Patients who received a CDK4/6i in any subsequent line of therapy	33 (16.7)	41 (35.0)
Palbociclib	17 (8.6)	32 (27.4)
Ribociclib	13 (6.6)	7 (6.0)
Abemaciclib	6 (3.0)	3 (2.6)

^a^Includes patients who received chemotherapy in combination with any non-chemotherapy

^b^Includes patients who received hormonal therapy + other therapy without chemotherapy

**Fig. 2:**
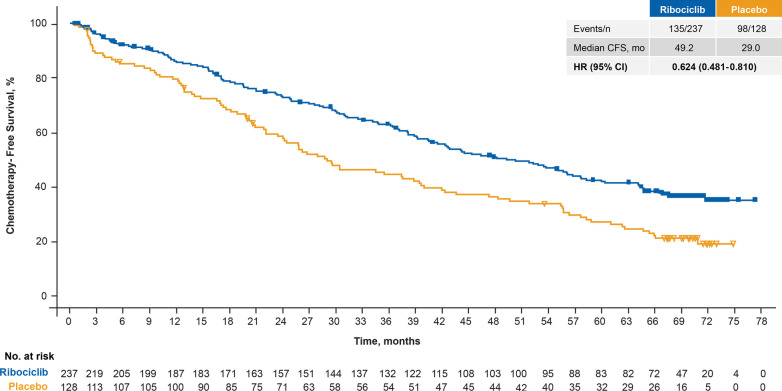
Chemotherapy-free survival in patients who received ribociclib plus fulvestrant as first-line therapy

The median CFS was 20.2 months longer: 49.2 (95% CI 40.5–57.7) months in the ribociclib arm versus 29.0 (95% CI 23.5–39.4) months in the placebo arm (HR 0.62; 95% CI 0.48–0.81) (Fig. 2). TTC was also delayed in patients receiving ribociclib versus placebo (mOS, NR vs. 42.0 months [HR 0.57; 95% CI 0.42–0.79]).

### PFS2 in patients in first-line subgroup

**Fig. 3:**
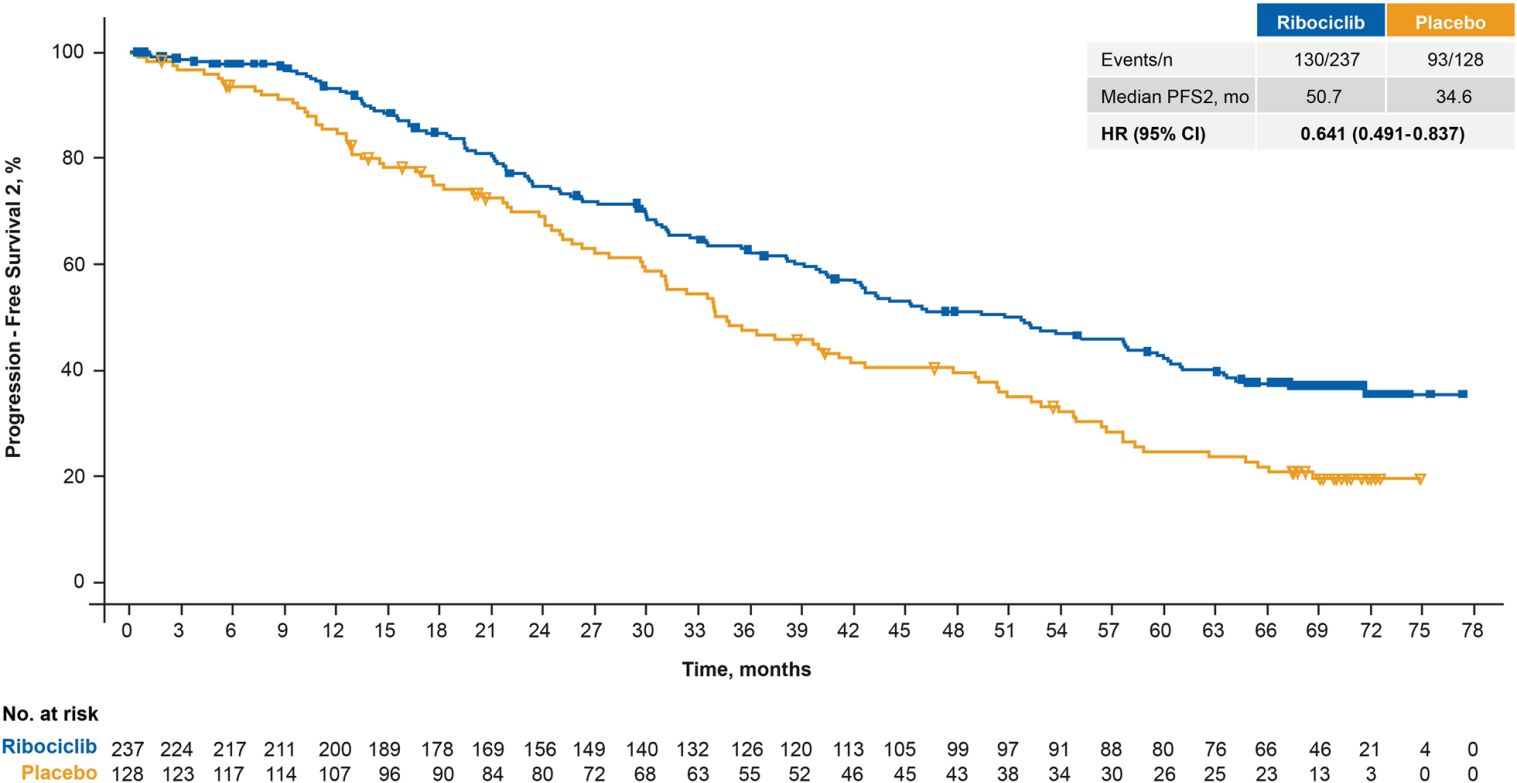
Progression free survival 2 in patients who received ribociclib plus fulvestrant as first-line therapy

Following study treatment in the first-line setting, 130 of 237 patients (54.9%) in the ribociclib arm and 93 of 128 patients (72.7%) in the placebo arm had disease progression while receiving subsequent therapy. The median PFS2 was 16.1 months longer in the ribociclib arm (50.7 months; 95% CI 42.1–58.9 months) versus the placebo arm (34.6 months; 95% CI 29.9–42.6 months [HR 0.64; 95% CI 0.49–0.84]; Fig. 3).

### Safety

With this extended follow-up analysis at 70.8 months, no new safety signals were observed. Overall, AEs for the overall trial population were consistent with those previously reported in the prior analyses of MONALESSA-3 [8, 17]. The most common grade 3/4 AE observed in the first-line patients (de novo or late relapse) was neutropenia (60.3% vs. 1.6% for ribociclib vs. placebo), with AEs of special interest being generally comparable to those previously reported for the overall trial population (Additional file 1: Table S2).

## Discussion

This exploratory analysis of MONALEESA-3, with a median follow-up of 70.8 months, reports the longest mOS benefit to date (67.6 months in the ribociclib + fulvestrant arm) for a first-line population (de novo or late relapse) in a phase III clinical trial setting in ABC. First-line ribociclib + fulvestrant demonstrated a nearly 16-month improvement in mOS versus fulvestrant alone, with a 33% relative reduction in the risk of death. Additionally, even though more patients in the placebo versus ribociclib arm (35.0% vs. 16.7%) received a CDK4/6i after discontinuing study treatment, patients in the ribociclib arm still experienced an OS benefit. These extended follow-up results clearly demonstrate the magnitude of survival benefit with ribociclib in the first-line setting, which was not fully revealed in the prior exploratory OS analysis of MONALEESA-3 for this subgroup [18]. The OS benefit in the overall trial population and the second-line subgroup (early relapse or one prior ET for ABC) was in line with the previously reported exploratory OS results, with a median 10.7-month and 6-month OS advantage over placebo, respectively [18]. Although this study was not designed to compare first- and second-line setting results, the HRs for the OS benefit with placebo versus ribociclib + fulvestrant were 0.67 and 0.80, respectively. Finally, no new safety signals were noted with nearly 6 years of follow-up, bolstering the evidence related to the safety profile of ribociclib treatment [8, 17, 18].

Postdiscontinuation observations are particularly helpful in providing additional insights into benefit beyond study treatment. The benefit of first-line ribociclib + fulvestrant was demonstrated after discontinuation of study treatment, with prolongation of median PFS2 by 16.1 months. Furthermore, median CFS was delayed by 20.2 months in patients receiving ribociclib in the first-line setting, with a 43% relative reduction in the risk of chemotherapy, compared with those receiving placebo. These postdiscontinuation results, along with the survival benefit demonstrated in MONALEESA-3, strengthen the efficacy profile and confirm the lasting benefit of first-line ribociclib treatment that extends well beyond study treatment. Recently the results of SONIA, which studied first-line NSAI + CDK4/6i followed by second-line fulvestrant versus first-line NSAI followed by second-line fulvestrant + CDK4/6i, were reported, with no significant difference in PFS2 or OS between the treatment arms [20]. However, it should be noted that the CDK4/6i used for 91% of the patients in SONIA was palbociclib. The relevance of these results may not apply to other CDK4/6is since palbociclib, unlike other approved CDK4/6is, has never demonstrated OS benefit in ABC. Additionally, the difference in outcomes for the respective phase III CDK4/6i trials in early breast cancer (no significant benefit for palbociclib [PALLAS, PENELOPE-B]; significant benefit for ribociclib [NATALEE], abemaciclib [MonarchE]) further suggest that results observed for palbociclib may not be relevant for the other approved CDK4/6is [21–26].

This is the third pivotal trial to demonstrate a significant improvement in OS with ribociclib in a first-line setting, and these data align with those of the MONALEESA-2 (first line in combination with letrozole in postmenopausal patients) trial and MONALEESA-7 (first line in combination with NSAI in pre/perimenopausal patients) trial, which demonstrated a 63.9- and 58.7-month mOS, respectively, in patients treated with ribociclib + ET [14, 16]. While all MONALEESA studies allowed patients treated in the first line, patients with early relapse were included for analysis in the second-line population of MONALEESA-3 and in the first-line population of MONALEESA-2/7 [14, 16, 17].

OS results with other CDK4/6is in combination with fulvestrant have been reported. However, it is important to understand the relevant differences in the patient populations of these studies to help put the data in context. MONALEESA-3 included a broad-spectrum patient population, in which first line was defined as either no prior treatment for breast cancer (de novo patients with no prior exposure to ET) or relapse > 12 months after (neo)adjuvant ET (late relapse) and second line was defined as one prior ET for ABC or relapse ≤ 12 months after the end of (neo)adjuvant therapy (early relapse) [17]. Both MONARCH-2 and PALOMA-3 enrolled patients previously treated with ET for ABC as well as those with early relapse. Patients with early relapse have a similar prognosis to those being treated in the second line, making the overall population of MONARCH-2 and PALOMA-3 similar to second-line populations (although early relapse remains in the first-line treatment setting). In MONARCH-2, the mOS was 46.7 months for abemaciclib + fulvestrant and 37.3 months for placebo + fulvestrant (HR 0.76; 95% CI 0.61–0.95; *P* = 0.01) [13]. In PALOMA-3, palbociclib + fulvestrant did not demonstrate a statistically significant improvement in OS versus fulvestrant alone (HR 0.81; 95% CI 0.64–1.03; *P* = 0.09) in the final prespecified analysis or in an extended follow-up exploratory analysis (HR 0.81; 95% CI 0.65–0.99) [11, 12]. Cross-trial comparisons should be interpreted with caution and are presented here to provide context to the results of the current analysis.

## Conclusions

In MONALEESA-3, with a follow-up of nearly 6 years, ribociclib + fulvestrant demonstrated the longest mOS observed to date for a first-line population in a phase III clinical trial. This is the third phase III study of ribociclib demonstrating a significant OS advantage in first-line treatment of patients with HR+/HER2− ABC. This analysis adds to the robust body of evidence on the efficacy of ribociclib use across the MONALEESA program and confirms its long-term, consistent OS benefit in treating patients with HR+/HER2− ABC irrespective of ET partner or menopausal status.

### Supplementary Information

**Additional file 1. Table S1**: Dispositions of patients who received ribociclib plus fulvestrant or placebo plus fulvestrant as first-line therapy. **Table S2**: Adverse events of special interest among patients treated with ribociclib plus fulvestrant or placebo plus fulvestrant as first-line therapy (safety set).

## Data Availability

Novartis made the study protocols available for MONALEESA-3 at the time of primary publications. Individual participant data will not be made available.
