# Supplementary material for: Updated overall survival from the MONALEESA-3 trial in postmenopausal women with HR+/HER2− advanced breast cancer receiving first-line ribociclib plus fulvestrant
Source: Breast Cancer Res. 2023 Aug 31;25:103. doi: 10.1186/s13058-023-01701-9 (PMC10469877; doi:10.1186/s13058-023-01701-9)
Supplement: Supplementary file 1 — Additional file 1. Table S1: Dispositions of patients who received ribociclib plus fulvestrant or placebo plus fulvestrant as first-line therapy. Table S2: Adverse events of special interest among patients treated with ribociclib plus fulvestrant or placebo plus fulvestrant as first-line therapy (safety set). [file 13058_2023_1701_MOESM1_ESM.pdf]

Supplementary Table 1. Dispositions of patients who received ribociclib plus fulvestrant or placebo plus fulvestrant as first-line therapy.

n (%)	Ribociclib + Fulvestrant n = 237	Placebo + Fulvestrant n = 128	All Patients N = 365
**Patients treated**			
Treatment ongoing[a]	39 (16.5)	11 (8.6)	50 (13.7)
End of treatment	198 (83.5)	117 (91.4)	315 (86.3)
**Reason for end of treatment**			
Progressive disease	133 (56.1)	101 (78.9)	234 (64.1)
Adverse event	23 (9.7)	5 (3.9)	28 (7.7)
Patient/guardian decision	22 (9.3)	4 (3.1)	26 (7.1)
Physician decision	19 (8.0)	6 (4.7)	25 (6.8)
Death	1 (0.4)	0	1 (0.3)
Protocol deviation	0	1 (0.8)	1 (0.3)
**Entered survival follow-up[b]**	167 (84.3)	110 (94.0)	277 (87.9)

[a] Patients continuing study treatment at the time of data cutoff (January 12, 2022). [b] The percentages of patients who entered survival follow-up used the number of patients with end of treatment as the denominator.

Supplementary Table 2. Adverse events of special interest among patients treated with ribociclib plus fulvestrant or placebo plus fulvestrant as first-line therapy (safety set).

n (%)	Ribociclib + Fulvestrant n = 237			Placebo + Fulvestrant n = 128		
**AESI grouping[a]**	**All grades**	**Grade 3**	**Grade 4**	**All grades**	**Grade 3**	**Grade 4**
Hematologic AESIs						
Neutropenia	175 (73.8)	123 (51.9)	20 (8.4)	6 (4.7)	2 (1.6)	0
Leukopenia	77 (32.5)	35 (14.8)	2 (0.8)	2 (1.6)	0	0
Anemia	40 (16.9)	6 (2.5)	0	12 (9.4)	2 (1.6)	0
Thrombocytopenia	16 (6.8)	0	0	3 (2.3)	0	0
Other	1 (0.4)	1 (0.4)	0	0	0	0
Nonhematologic AESIs						
Infections	146 (61.6)	21 (8.9)	0	65 (50.8)	6 (4.7)	0
Hepatobiliary toxicity	63 (26.6)	26 (11.0)	6 (2.5)	22 (17.2)	5 (3.9)	0
Renal toxicity	30 (12.7)	2 (0.8)	0	10 (7.8)	0	0
QT interval prolongation	25 (10.5)	12 (5.1)	0	1 (0.8)	1 (0.8)	0
ILD/ Pneumonitis	8 (3.4)	2 (0.8)	0	1 (0.8)	0	0
Reproductive toxicity	1 (0.4)	0	0	1 (0.8)	0	0

AESI, adverse event of special interest; ILD, interstitial lung disease.
[a] Patients with multiple events in a grouping are counted only once in the grouping under the maximum grade.
